# Expressions of ion co-transporter genes in salicylate-induced tinnitus and treatment effects of spirulina

**DOI:** 10.1186/s12883-016-0682-4

**Published:** 2016-09-02

**Authors:** Juen-Haur Hwang, Yin-Ching Chan

**Affiliations:** 1 Department of Otolaryngology, Dalin Tzu Chi Hospital, Buddhist Tzu Chi Medical Foundation, Chiayi, Taiwan; 2 School of Medicine, Tzu Chi University, Hualien, Taiwan; 3 Department of Food and Nutrition, Providence University, Taichung, Taiwan

**Keywords:** Tinnitus, Salicylate, mRNA, K^+^– Cl^−^ co-transporter gene, Na-K-Cl co-transporter gene, Spirulina

## Abstract

**Background:**

Although the activity of tinnitus-related ion co-transporter are known, their mRNA expressions has seldom been reported. We aimed to investigate the mRNA expressions of tinnitus-related ion co-transporter genes, and treatment effects of Spirulina.

**Methods:**

The mRNA expressions of K^+^–Cl^−^ co-transporter (KCC2) and Na-K-2Cl co-transporter 1 (NKCC1) genes in the cochlea and brain of mice were evaluated after tinnitus was induced by intraperitoneal injection of salicylate. The effects of spirulina water extract on these gene expressions were investigated.

**Results:**

Compared to the control group, the tinnitus scores increased significantly, however, the salicylate-induced tinnitus could be reduced significantly by spirulina water extract. The tinnitus group had higher of borderline significance mRNA expression of KCC2 gene in the cochlear, significantly higher in the temporal lobes and in the frontal lobes. Meanwhile, compared to the tinnitus group, the spirulina group had significantly lower mRNA expression of KCC2 gene in the cochlear, temporal lobes, frontal lobes and parahippocampus/hippocampus. However, the NKCC1 mRNA expression was not significantly different between three groups in the cochlea and these brain areas.

**Conclusion:**

Salicylate-induced tinnitus might be associated with increased mRNA expression of KCC2 gene, but not with mRNA expressions of NKCC1 gene in the cochlear and some tinnitus-related brain areas. Spirulina reduced the expression of KCC2 genes in salicylate-induced tinnitus.

## Background

Subjective tinnitus is most commonly believed to originate from the cochlear and/or central nervous system dysfunction at the levels of the brainstem, subcortical and cortical level, as well as the limbic system [1, 2]. The functions of certain neurotransmitter receptors, ion channels and/or co-transporters have been proposed to be associated with tinnitus, for example, activation of vanilloid receptors in the inner ear [3], N-methyl D-aspartate receptor (NMDA receptor, NR) in the inner ear [4], acetylcholine receptors in the auditory cortex [5], and dopamine receptors in the auditolimbic dopaminergic pathway exhibited excitatory effects on tinnitus [6]. On the contrary, activation of cannabinoid receptors in the ventral cochlear nucleus [7] and γ-amino butyric acid receptors (GABA receptor, GR) in the inferior colliculus (IC) [8] or auditory cortex [9] exhibited inhibitory effects on tinnitus.

Some co-transporter and/or ion channels, for example, K^+^– Cl^−^ co-transporter (KCC2) [10, 11], voltage-gated sodium channels [12, 13], transient outward potassium currents, the glycine-induced chloride channels [14], L-type calcium channels [15], and Na-K-2Cl co-transporter 1 (NKCC1) [16], were reported to be associated with tinnitus. Among them, KCC2 is a neuron-specific Cl^−^ transporter whose role in adult central nervous neurons is to maintain low intracellular Cl^−^ concentrations needed for the hyperpolarizing responses to the inhibitory amino acid GABA and glycine of the auditory nucleus [10, 17]. The NKCC is a class of membrane proteins that transport Na, K, and Cl ions into and out of a wide variety of epithelial and non-epithelial cells. The NKCC1 isoform is present in a wide variety of tissues, whereas, NKCC2 is found only in the kidney [18]. Inhibition of NKCC1 with its potent antagonist bumetanide reduces pain behavior in rats following spinal cord injury [19].

Tinnitus-related changes in gene expression have also played an important role in tinnitus. For example, the mRNA expression of the c-fos gene increased in the auditory brainstem [20] and cortices [21]. The expression of NR subtype 2A (NR2A) genes increased in the auditory cortices of rats in salicylate-induced tinnitus [21]. The expression levels of NR2B, tumor necrosis factor-α (TNF-α) and interleukine-1β (IL-1β) genes increased significantly, whereas cyclooxygenase type 2 (COX-2) gene expression decreased in the cochlea and IC of mice with salicylate-induced tinnitus [22, 23]. NKCC1 mRNA expression increased in the chronic tinnitus and the recovery period, but decreased in the phase of acute tinnitus [16]. However, the mRNA expression of KCC2 was still unclear in tinnitus. Furthermore, based on recent animal studies in our laboratory, neural inflammation and oxidation-reduction status (redox) may be novel mechanisms in tinnitus [22–24]. Meanwhile, it is well known that inflammation and/or oxidative stress could violate cell functions not only at the cell membrane level, but also at the cytoplasm and nucleus levels. Thus, it is reasonable to expect that anti-inflammatory and/or anti-oxidative agents might prevent cell dysfunction in genetic level and beyond.

Spirulina is a microscopic blue-green alga. Spirulina platensis and its active ingredient, C-phycocyanin (CPC), exert antioxidative, anti-inflammatory effects via inhibition of COX-2 [25] and/or nicotinamide adenine dinucleotide phosphate (NADPH) oxidase enzymes [26]. S. platensis water extract significantly slowed the loss of memory by augmenting catalase (CAT) activity in the brain of the senescence- accelerated prone-8 (SAMP8) mouse sub-strain [27]. CPC and S. platensis water extract reduced salicylate-induced tinnitus and down-regulated mRNA and protein expression for NR2B, TNF-α, IL-1β, and COX-2 genes in the cochlea and IC of SAMP8 mice [24]. It reduced also salicylate-induced over-expression of the manganese-superoxide dismutase (Mn-SOD) gene and malondialdehyde (MDA) levels, but increased salicylate-induced down-regulation of the CAT gene in many brain areas [28]. However, the effects of S. platensis water extract on the mRNA expression of KCC2 and/or NKCC1 genes after salicylate-induced tinnitus have not so far been reported.

As we have mentioned above, the functions of KCC2 and NKCC1 were associated with tinnitus. But, the mRNA expression of these co-transporter genes in salicylate-induced tinnitus and the effects of S. platensis water extract on them were still limited or unclear. Therefore, we aimed to investigate these questions. We hypothesized that mRNA expression of KCC2 and/or NKCC1 genes altered after 4-days of salicylate-induced tinnitus in the cochlea and brain. S. platensis water extract could modulate on the expression of KCC2 and/or NKCC1 genes after salicylate-induced tinnitus.

## Methods

### Animals

Twenty-four 3-month-old male SAMP8 mice were randomly and equally divided into three groups (8 mice in each group): a control group (saline-treated), tinnitus group (salicylate-treated), and spirulina group (salicylate plus S. platensis water extract-treated). All groups were fed with normal diet. Institutional Animal Care and Use Committee of Dalin Tzu Chi Hospital approved the protocol used in this study.

### Behavioral conditioning to active avoidance task

All mice were trained to perform an active avoidance task, which was performed in a conditioning box with an electrical floor and a climbing pole, according to the design of Guitton et al. [29] and Hwang et al. [22–24].

The conditioning paradigm consisted of six sessions performed per day for five days (day 1–5). Each session lasted 15 to 20 min, as there were 10 trials per session. Inter-trial intervals lasted at least one minute. For each trial, the conditioning stimulus was a 50 dB sound pressure level (SPL) pure tone with a frequency of 10 kHz and of a three-second duration, and the unconditioned stimulus was a 3.7 mA electric foot-shock presented for 30 s, re. Guitton’s protocol [29], by adjusting the electric voltage with fixed copper wire resistance on the floor. The time between the conditioned stimulus and the unconditioned stimulus was one second. The mice would climb up through the pole into a safe area after the coupled conditioned and unconditioned stimuli. Electrical shocks were stopped by the experimenter when the animal climbed correctly. The “true-positive” score was the level of performance assessed by the number of times the mice climbed correctly in response to sound. Mice were considered to be conditioned when the “true-positive” score reached at least 80 % in three consecutive sessions. Only conditioned mice were placed in the tinnitus experiments.

### Induction and testing of tinnitus

When conditioned, an active avoidance task of one session with 10 trials was performed 2.0 h after intraperitoneal injections of saline, either alone or containing 300 mg/kg sodium salicylate (Sigma - Aldrich, St. Louis, MO) for 4 days (day 6 ~ 9). To avoid changes attributable to hearing loss induced by salicylate (about 10 to 20 dB during 4-days injections), the intensity of the sound that elicited the behavioral responses was adjusted by increasing the sound intensity to 70 dB SPL for the salicylate-treated group. By doing so, we could keep the sound sensation levels of all mice in three groups almost similar [22–24].

During tinnitus testing, a sound of three-seconds duration was given first for 3.0 s in each trial, and the mice were observed for another 5.0 s to check if they would perform the task correctly (true-positive). If so (true-positive), the mice were put down on the floor for ongoing observation, i.e., if animals stayed on the safe area >10 s. If not, electrical shock was given by the experimenter to remind the mice to climb up the pole. Then, the mice were also placed on the floor for ongoing observation, i.e., if animals stayed on the safe area >10 s.

Finally, the experimenter observed the total number (false-positive score or tinnitus score) of times the mice climbed during the inter-trial silent period of 1.0 min composed of 10 trials.

### Samples isolation and RNA extraction from the cochlea and brain

The pairs of cochlea, brainstem including the IC, temporal lobes, frontal lobes, and para-hippocampus/hippocampus were immediately dissected using a Zeiss stereomicroscope and stored at −80 °C refrigerator until used. RNA isolation was performed using the RNA-Bee isolation reagent (Friendswood, USA) with a tissue homogenizer according to the manufacturer’s protocol. The RNA quality was assessed by an Agilent Bioanalyzer 2100, and the ratio of absorbance measurements was conducted at 260 and 280 nm using the nanodrop method.

### Reverse transcription-polymerase chain reaction (RT-PCR)

cDNA was synthesized from total RNA by reverse transcription using MMLV high performance reverse transcriptase (Epicentre Biotechnologies, USA) in a P × 2 Thermal cycler (Thermo Electron Corporation Bioscience Technologies Division, USA). For each RT reaction, a positive control was performed with 1.0 μg of total RNA. RT was carried out at 37 °C for 1 h. For PCR amplification, 3.0 μL cDNA and primers were used according to the supplier’s instructions with DreamTaq™ DNA polymerase (Fermentase, USA). The primers were: KCC2-F-5′-CTCAAC AACCTGACGGACTG-3′, KCC2-R-5′-GCACAACACCATTGGTTGCG-3′ for brain tissues [30]; KCC2-F-5′-ATGTACATCCTTGGCACGAT -3′, KCC2-R-5′-AGGAAGACC AAGGCAAACTT-3′ for cochlear tissues [31]; NKCC1-F-5′-TGT TGGATTCGC AGAGACTG-3′, NKCC1-R-5′-GTTCCTTTGGGTATGGCTGA-3′ [32]; β-actin F-5′-CCACACCCGCCACCAGTTCG-3′, β-actin R-5′-CCCATTCCC ACCATC ACACC-3′ [33]. The thermal cycling conditions for PCR were adjusted to the following: 3.0 min initial set-up at 95 °C; followed by 50 cycles, each of which consisted of 45 s of denaturation at 95 °C, 45 s of annealing at 56 °C, and 72 s of extension at 72 °C for KCC2 gene of brain tissues; 45 s of denaturation at 95 °C, 45 s of annealing at 50 °C, and 72 s of extension at 72 °C for KCC2 gene of cochlear tissues; of 45 s of denaturation at 95 °C, 45 s of annealing at 52.1 °C, and 72 s of extension at 72 °C for NKCC1 gene; of 45 s of denaturation at 95 °C, 45 s of annealing at 50 °C, and 72 s of extension at 72 °C for β-actin gene. A final 10 min at 72 °C for all above genes.

### Quantitation of PCR products

The DNA products were measured by the Mini Horizontal Electrophoresis System (MJ-105/MP-100, Major Science, Taiwan) and by E-Box-1000/26 M Inspection Certificate & Analysis Systems (E-Box Spp-010 E-capt soft ware, USA). The expression levels of KCC2 and NKCC1 genes were presented as relative ratios in comparison to β-actin gene.

### Statistical analysis

The data are presented as the mean ± standard deviation (SD), unless indicated otherwise. The tinnitus scores were compared between the three groups by a one-way ANOVA test. The mRNA expression levels of KCC2, NKCC1 or β-actin genes at each site were compared also by one-way ANOVA test. All above analysis were performed by the commercialized software “STATA10”, and *p* values <0.05 were considered statistically significant.

## Results

**Fig. 1:**
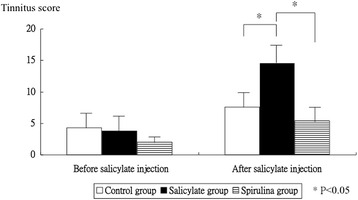
The tinnitus scores before and after salicylate injection. The tinnitus score was not significantly different in three groups before salicylate injection. However, after salicylate injection, the tinnitus group had higher significantly tinnitus score than the control group, and the spirulina group had significantly lower tinnitus score than the tinnitus group

Figure 1 display the tinnitus scores before, and 4.0 days after salicylate injection. The tinnitus score was not significantly different in three groups before intraperitoneal salicylate injection (4.3 ± 2.9 versus 3.8 ± 2.9 versus 2.0 ± 1.0, *p* = 0.3318). But, the tinnitus score was significantly different in three groups four days after intraperitoneal salicylate injection (7.6 ± 3.8 versus 14.5 ± 4.6 versus 5.4 ± 3.4, *p* = 0.0005). The tinnitus group had a higher significant tinnitus score than the control group (*p* = 0.006); whereas the spirulina group had significantly lower tinnitus score than the tinnitus group (*p* = 0.001).

**Fig. 2:**
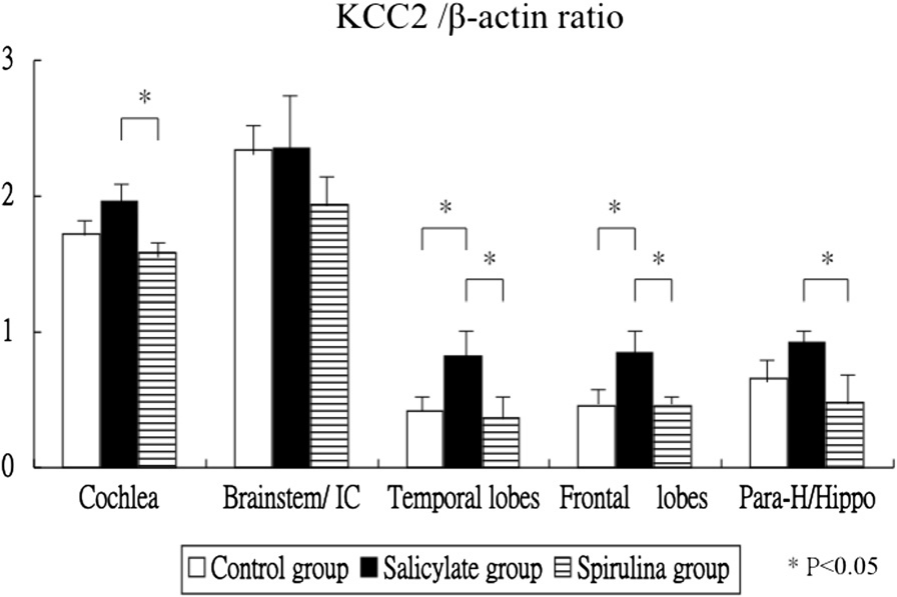
The mRNA expression levels of the KCC2 gene. The KCC2 expression was significantly different between three groups in the cochlea, temporal lobes, frontal lobes, and in the hippocampus/parahippocampus, but of borderline significance in the brainstem

Figure 2 shows the mRNA expression levels of the KCC2 gene in the cochlea and several brain areas. The KCC2 mRNA expression was significantly different between three groups in the cochlea (one-way ANOVA, *p* = 0.0036). The tinnitus group had higher of borderline significance mRNA expression of KCC2 gene than the control group in the cochlea (1.96 ± 0.22 versus 1.72 ± 0.13, *p* = 0.066); whereas the spirulina group had significantly lower mRNA expression of KCC2 gene than the tinnitus group in the cochlea (1.58 ± 0.12 versus 1.96 ± 0.22, *p* = 0.003). Second, the KCC2 mRNA expression was different and of borderline significance between the three groups in the brainstem (2.34 ± 0.28 versus 2.36 ± 0.43 versus 1.94 ± 0.30, one-way ANOVA, *p* = 0.0937). Third, the KCC2 mRNA expression was significantly different between the three groups in the temporal lobes (one-way ANOVA, *p* = 0.0067). The tinnitus group had significantly higher mRNA expression of KCC2 gene than the control group in the temporal lobes (0.82 ± 0.24 versus 0.42 ± 0.19, *p* = 0.025), and the spirulina group had significantly lower mRNA expression of KCC2 gene than the tinnitus group in the temporal lobes (0.37 ± 0.25 versus 0.82 ± 0.24, *p* = 0.010). Fourth, the KCC2 mRNA expression was significantly different between three groups in the frontal lobes (one-way ANOVA, *p* = 0.0008). The tinnitus group had significantly higher mRNA expression of KCC2 gene than the control group in the frontal lobes (0.85 ± 0.23 versus 0.46 ± 0.12, *p* = 0.003), and the spirulina group had significantly lower mRNA expression of KCC2 gene than the tinnitus group in the temporal lobes (0.45 ± 0.11 versus 0.85 ± 0.23, *p* = 0.002). Fifth, the KCC2 mRNA expression was significantly different between three groups in the para-hippocampus/hippocampus (one-way ANOVA, *p* = 0.0074). The tinnitus group did not display significantly higher mRNA expression of KCC2 gene than the control group in the hippocampus/parahippocampus (0.92 ± 0.11 versus 0.66 ± 0.23, *p* = 0.143), but the spirulina group had significantly lower mRNA expression of KCC2 gene than the tinnitus group in the parahippocampus/hippocampus (0.48 ± 0.25 versus 0.92 ± 0.11, *p* = 0.006).

**Fig. 3:**
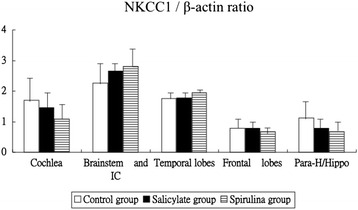
The mRNA expression levels of the NKCC1 gene. The KCC2 expression was not significantly different between three groups in all tested areas

Figure 3 shows the mRNA expression levels of the NKCC1 gene in the cochlea and several brain areas. The NKCC1 mRNA expression was not significantly different between the three groups of the cochlea (1.70 ± 0.89 versus 1.45 ± 0.60 versus 1.08 ± 0.64, one-way ANOVA, *p* = 0.3469), in the brainstem (2.26 ± 0.82 versus 2.66 ± 0.20 versus 2.80 ± 0.80, one-way ANOVA, *p* = 0.3714), in the temporal lobes (1.75 ± 0.23 versus 1.78 ± 0.19 versus 1.94 ± 0.12, one-way ANOVA, *p* = 0.1991), in the frontal lobes (0.78 ± 0.20 versus 0.78 ± 0.17 versus 0.68 ± 0.15, one-way ANOVA, *p* = 0.5237), and in the parahippocampus/hippocampus (1.13 ± 0.61 versus 0.87 ± 0.32 versus 0.95 ± 0.30, one-way ANOVA, *p* = 0.5802).

## Discussion

This experimental study showed that mRNA expression of KCC2 gene increased, whereas NKCC1 gene expression did not change significantly, in the cochlea and several tinnitus-related brain areas during salicylate-induced tinnitus. Combined with previous reports about the changes of ion channel and/or co-transporter functions [10, 11, 18, 19], we suggested that a high dose of salicylate could not only induce tinnitus, but also could modulate expressions of KCC2 in the genetic level and beyond in mice. However, our results related to NKCC1 gene expression were not consistent with findings of Yao et al. [16], in which the NKCC1 mRNA expression increased in the chronic tinnitus phase and the recovery period, but decreased in the acute tinnitus phase.

Early expression of the KCC2 selectively enhances GABAergic synapses [34]. Targeted deletion of the neuronal-specific KCC2 generates mice with a profound seizure disorder and confirms the central role of this transporter in modulating neuronal excitability [35], whereas the KCC2 could promote GABAergic excitation in the mature rat hippocampus during ictal epileptiform activity [11]. K-Cl co-transporter KCC3 may be involved in degenerative peripheral neuropathies [19]. The neuronal KCC2 maintains the low intracellular chloride concentration required for the fast hyperpolarizing actions of inhibitory neurotransmitters in the mature central nervous system (CNS) [10, 17]. We found that salicylate could increase the mRNA expression of KCC2. However, the exact mechanisms linking this finding and salicylate-induced tinnitus remain further studies.

The renal-specific Na-Cl co-transporter (NCC) and Na-K-2Cl co-transporter (NKCC2) are involved in Gitelman and Bartter syndrome, respectively, and the autosomal recessive forms of metabolic alkalosis. NKCC1 is involved in hearing, salivation, pain perception, spermatogenesis, and the control of extracellular fluid volume. Inhibition of NKCC1 with its potent antagonist bumetanide reduces pain behavior in rats following spinal cord injury [19]. NKCC1 mRNA expression increased in the chronic tinnitus and recovery period, but decreased in the acute tinnitus condition [16]. However, the expressions of NKCC1 gene were not altered significantly in the cochlea and several tinnitus-related brain areas investigated in our study. This discrepancy might be due to the differences in study design, dose of salicylate, interval between the entry and the end of study, etc.

The inflammation and/or redox imbalance might interfere with the neural cell functions via modulation of ion channels, cotransporters, and/or neurotransmitters. In addition, inflammation might alter mRNA expression of cation chloride co-transporters, including KCC2 and NKCC1, in sensory neurons [36–38]. In acute arthritis, both NKCC1 and KCC2 mRNA increased in superficial dorsal horn, and this was accompanied by an increase in protein expression. In chronic arthritis, NKCC1 expression remained raised, but KCC2 mRNA and protein expression returned to control levels [36]. Furthermore, based on results from recent animal studies in our laboratory, neural inflammation [22, 23] and oxidative stress [24] may be novel mechanisms in tinnitus. We had found that spirulina water extract, with its ant-inflammatory and anti-oxidative abilities [25, 26], could reduce salicylate-induced tinnitus and up-regulation of TNF-alpha, IL-1beta, NR2B, and COX-2 [22, 23]. In this study, we found that spirulina could reduce the salicylate-induced up-regulation of KCC2 gene in the cochlea, temporal lobes, frontal lobes, and para-hippocampus/hippocampus. Thus, we proposed that spirulina might modulate the mRNA expression of KCC2 by reducing neuroinflammation and/or oxidative stress. However, we indeed could not know clearly if the effect of Spirulina was to antagonize the effect of salicylate and/or was on the basal expression of these transporters.

## Conclusion

Salicylate-induced tinnitus might be associated with increased mRNA expression of KCC2 gene, but not with mRNA expression of NKCC1 gene in the cochlea and tinnitus-related brain areas. This animal study provided new information that high doses of salicylate could not only induce tinnitus, but also could modulate expressions of KCC2 and NKCC1 at the genetic level and beyond in mice. In addition, spirulina water extract might modulate the mRNA expression of KCC2 possibly by reducing neuroinflammation and/or oxidative stress.

## Abbreviations

CAT: Catalase
COX-2: Cyclooxygenase type 2
CPC: C-phycocyanin
GR: γ-amino butyric acid receptor
IL-1β: Interleukine-1β
KCC2: K^+^– Cl^−^ co-transporter
NADPH: Nicotinamide adenine dinucleotide phosphate
NKCC1: Na-K- 2Cl co-transporter 1
NR: N-methyl D-aspartate receptor
SAMP8: Senescence-accelerated prone-8
SPL: Sound pressure level
TNF-α: Tumor necrosis factor-α
